# Live Opera Performances on Veteran Recovery from Homelessness and Post Traumatic Stress: Post- Discussion Qualitative Analysis

**DOI:** 10.1007/s10597-025-01532-2

**Published:** 2025-11-13

**Authors:** Kia Skrine Jeffers, Tiffany Dzou, Robert M. Bilder, Joseph D. Mango, Jose Pila, Kenneth Wells

**Affiliations:** 1 University of California Los Angeles, School of Nursing, Los Angeles, CA USA; 2 Smidt Heart Institute, Cedars-Sinai, Los Angeles, CA, USA; 3 Jane & Terry Semel Institute for Neuroscience and Human Behavior and Department of Psychiatry and Biobehavioral Sciences, David Geffen School of Medicine and Department of Psychology, UCLA College of Letters and Science, University of California Los Angeles, Los Angeles, CA USA; 4 Division of Behavioral & Organizational Sciences, Claremont Graduate University, Claremont, CA USA; 5 RAND Corporation, Santa Monica, CA USA; 6 Department of Health Policy and Management, University of California Los Angeles, Fielding School of Public Health, Los Angeles, CA USA; 7 Greater Los Angeles Veterans Administration Health System, Los Angeles, CA USA

**Keywords:** Veterans, Stress disorders, Post-Traumatic, Mental health, Housing instability, Resilience, psychological, Creative arts, Opera

## Abstract

There is limited research on the impact of art forms, including opera, on audience and cast engagement in understanding mental health needs and support for Veterans at risk for post-traumatic stress disorder (PTSD) and homelessness. This qualitative study explores post-performance discussions from audience and cast members after live performances of an opera about Vietnam Veterans and family members, and their experiences of war-time trauma, housing instability, and receiving services to promote recovery and resilience. Audio-recorded and transcribed comments by Veterans (*n*=8), family members (*n*=9), providers (*n*=5) and cast (*n*=9) were analyzed using thematic analysis for key themes, with examples of quotes. Main themes included: 1) *Struggling Post-military Service*, including feeling misunderstood and self-isolation; 2) *Finding Hope*, through commitment and community; and 3) *Healing through the Opera* by sharing difficult societal issues, promoting empathy, and sharing resources, with lived experiences shared across themes by Veterans, family members, providers and cast/production team with a peer facilitator. The reflections they shared about their journey from trauma and homelessness through implementing strategies to obtain needed services, were fostered through live opera performances and peer-facilitated, post-discussions.

## Introduction

Creative arts can foster compassion and understanding towards people with mental illness and social problems like unstable housing (Mango et al., 2018) and increase collective awareness and commitment to improving mental health care (Chung et al., 2009). There are limited data on the impact of opera as an art form, as literature primarily focuses on singing or musical training leading to positive affect (Grape et al., 2002; Man et al., 2021; Sandgren, 2009; Thomson et al., 2017). Opera is considered a complex art form of interest to clinical psychology (Cohen, 2009), which may reduce social stigma of grief and schizophrenia (Skrine Jeffers et al., 2022). Studies comment on the influence of music and staging (Boerner et al., 2008; Simonton, 2000), but less is known about the impact of live performances on audiences’ knowledge and attitudes towards mental health (Grape et al., 2002). Scherer et al. (2019a, 2019b) identified three routes of impact from operas: (1) Structural aspects of lyrics and music; (2) performance aspects; and (3) listener characteristics. Differences may exist between online and live performances (De la Vega et al., 2020).

Veterans are at high risk for housing instability and post-traumatic stress disorder (PTSD) (Friedman et al., 1994; Lucas et al., 2021; Montgomery et al., 2021; Tsai & Rosenhack, 2015). Furthermore, these issues are stigmatized and limit access to treatment (Ijadi-Maghsoodi et al., 2021). There is limited research on the impact of creative arts in increasing public understanding of Veterans in the journey to services and recovery (Fancourt & Finn, 2019; Skrine Jeffers et al., 2022).

We reported on developing the “Veteran Journeys” opera using interviews of Veterans and family members and personal experiences of the psychiatrist-composer to describe experiences of Veterans with homelessness and PTSD (Wells, 2022; Wells et al., 2023). Due to physical distancing restrictions due to the COVID-19 pandemic, pre-recorded performances were presented via Zoom productions. From pre- and post-surveys, Zoom chat comments and emails from attendees, the findings indicated a pre-post increase in audience willingness to engage with Veterans with PTSD or unstable housing (Bilder et al., 2022), including among attendees with lived experience with trauma or unstable housing. Further, evidence suggested positive affect and social connectedness after the opera. Comments in the Zoom chat reflected emotional connections that audience members experienced, concerns about witnessing trauma/abuse, interest in receiving feedback from Veterans, and recommendations for a Veteran host to lead future post-performance discussions. In response, we produced in-person performances at an academic auditorium with Veterans brought from a local Veterans Administration Health System, and the post-performance discussions were facilitated by a Veteran peer specialist. This paper describes the experience of live performance through peer-facilitated discussion on Veteran, family, provider attendees and cast/production members.

## Methods

### Study Design and Sample

This study utilized a qualitative descriptive design (Sandelowski, 2000), approved by RAND human subjects review, w2812-98-01. Audiences were invited to attend through social media and email invitations from production members, UCLA, and the Greater Los Angeles Veterans Administration (VA). Bus services were provided for Veterans for the second performance. A Veteran host invited audience, cast, and production members to discuss experiences after watching the opera. The composer read the oral consent, noting that participation was voluntary and that the recorded discussion would be transcribed and de-identified for analysis.

### Researcher Positionality

The authors acknowledge the potential influence that several members of the research team had on the study given their role in the production and post-performance discussions. Authors include both the composer (KBW) and the Veteran facilitator of the post-performance discussion (JP), and another author (JDM) who assisted with disseminating invitations to the events. The lead analyst/author (KSJ) was not directly involved in production activities. Although all authors reviewed the analysis findings, the final integration and interpretation were conducted by the lead author (KSJ). The evaluation findings may shape the authors’ views of the arts’ contributions to mental health, potentially influencing subsequent events and related works.

### Data Collection

Data were collected through live discussions facilitated by an Army Veteran with Iraq and Afghanistan combat experiences that were “unfortunately not that different from” the opera. He noted he was “a service provider and started a nonprofit to assist Veterans with housing.” He explained: “After my military career and an honorable discharge, I ended up homeless. PTSD among other things led me to homelessness.” The facilitator projected a discussion slide (Table 1) that was developed using an adapted socioecological framework (internal, interpersonal, external factors) for audiences to reflect on their experiences to art events (Mango et al., 2018; Salihu et al., 2015). Discussions lasted 30 min.

**Table 1:** Post-Performance Discussion Guide

1. For Veterans here and family members – what does the opera mean to you? Probe: How has this affected you? Does it seem relevant to your experiences? Probe: What do you think the opera says about the experience of Veterans and family members?
2. For other audience members and providers, any comments on what the opera means to you? Probe: What does the opera say about the experience of providers? Probe: What are your thoughts about how the arts can affect our views of the experience of trauma fromwar or social problems such as housing insecurity? Probe: Other comments?
3. For the cast, what has the experience of performing the opera meant to you?
4. For creator of the opera, can you tell us something about the background of how and why this opera was made? Probe: What does it mean to you?

Questions and probes invite reflection on intrapersonal, interpersonal, and contextual/societal factors for audience members and cast, with the peer Veteran facilitator also sharing his experience

### Data Analysis

In keeping with qualitative descriptive design (Sandelowski, 2000), we employed a low-inference analytical approach where we stuck closely to participants’ narratives to fundamentally describe their experiences. The six phases of thematic analysis (Braun & Clarke, 2022) were used as a structured approach to analyze data from the post-performance discussion transcripts: (1) reviewing data; (2) coding; (3) generating initial themes; (4) reviewing themes; (5) refining, defining and naming themes; (6) writing up, by 3 investigators in stages. Themes and sub-themes were identified by similar phrases, patterns or sequences of ideas and feelings within and across groups (Table 2).

**Table 2:** Themes from audience and cast feedback in Post-Discussion with illustrative quotes (P1 = post discussion 1; P2 = post discussion 2)

Theme/Subtheme	Illustrative Quotes (Audience and cast comments post-opera)
Theme 1: Struggling post-military service	• It (opera) brings to life everything we struggle with; no matter how long you’ve been home, we constantly struggle with what being in combat, being in war, did. (P1). • After separating from the military. It took me to some pretty dark places, often in homelessness and in substance abuse. (P2).
Subtheme: Feeling misunderstood	• It’s tough about getting out and don’t mention it, and trying to find that groove, I didn’t think I got out of the military, not that I resented or hated my military service; I resented it because I thought it turned me into this beast that a lot of people saw. (P1) • No matter how long you’ve been home, we constantly struggle with what being in combat, being war, did; it’s a bitch really, people don’t understand what you have to keep doing in order to (have) Some Enchanted Evening. (P1)
Subtheme: Self isolating behavior	• I think that the thing of him (opera character) saying I want to be alone, I need to be alone, but I hate to be alone, that really resonated with me; like I mentioned with the scene with my ex-wife, I pushed her away, I felt like I had to get out of there and do everything to isolate from everyone. (P1) • Unfortunately, our friends and former veterans don’t take advantage of (resources); they want to isolate, to be alone, it’s not the way to heal. (P1) • Survivor’s guilt, similar to PTSD, just wanted to touch that a lot of veterans isolate themselves too from other vets because of that. (P1)
Theme 2: Finding Hope Subtheme: Commitment and Understanding	• The powerful message that a lot of people who are not married to a Veteran, the scene brought me to tears with the 2 of them, with all that she went through with him, most would have left long ago; when you don’t leave because you do see the light at end of that tunnel and you know who that person is, it’s very hard for other people to understand why you hang in there; I loved how that was presented; it’s very important for people to understand. (P1) • I just want to say thank you for bringing awareness to what we do go through in recovery on a personal level for Veterans and our families it’s a real relief to know that you do understand and are sharing it with community. (P2) • I’m on the road to recovery, to build a new career, because the hospitality career is over. So I’ll build a new career where I’ll be able to take those dark experiences and translate them for therapists as a peer support to let them know to help Veterans that are still struggling and everything like that; that’s where I am in my journey and this is like right on time. Tuesday is like a send-off in my next step, in my journey and thank you very much because I really appreciated the entire show. (P1)
Subtheme: Finding Community	• What I found in all of that, coming here though was finding community, and looking for community and finding support system and learning how to ask for it; learning the right way to ask for it too, because sometimes people who aren’t going through trauma hear the question, but they don’t register the trauma that you’re going through. And what I appreciated about this is that it really is about community and being aware of the people around you and knowing who you can go to and understanding that there are resources out there but you do have to sometimes ask for them, because they’re not just necessarily going to find you, but when you make that step they will start finding you. (P2)
Theme 3: Healing through the Opera	• There’s no like sugar coating of what Veterans have been through; and I think that honoring the service and honoring the experience of what happens when you return through music can aid in healing; that’s how this piece has been serving me as I’ve been going through my own experience and singing it, the new experience and I find ways to key into stuff. (P1) • What I found in all of that, coming here though was finding community, and looking for community and finding a support system and learning how to ask for it; learning the right way to ask for it too, because sometimes people who aren’t going through trauma hear the question but they don’t register the trauma that you’re going through. (P2) • Marrying two of my passions, making an impact and also singing. (P2). • You hear a pretty song, you think it spoke to the problem. We can help as artists. (PI) • If you can heal through art, beauty and song and story—I just love that we may be able to contribute to that through telling this story. (P2) • I recorded this during the lock down, right when we were going through all of our collective trauma together. I didn’t really absorb any of the material in full, honestly. And performing it now live is a completely different experience. (P2)
Subtheme: Addressing difficult societal issues among Veterans	• To be able to sing an opera that is about something (PTSD) similar to what I’m going through, for people to be able to see it […] this is a way to bring forth more healing, especially when through the use of art, if that speaks to you […] something that is so brutally honest, and setting it to music in such a beautiful way, that it touches you it affects you. (P2) • I’ve spent some time with Veterans and have deep respect for the service and the experience that you all have been through; and it’s a huge responsibility for us as a society to honor that and also to help heal what it is, whether it’s singing a song, you hear a pretty song, you think it spoke to the problem (PTSD). (P1)
Subtheme: Enhancing empathy towards Veterans and military families	• I just wanted to say that this opera helped me open my eyes to see what Veterans had gone through and what they experienced, and stuff that we don’t see, that our family members have gone through those dark places; I just wanted to say that this opera was amazing, that I liked the fact that it was storytelling through songs. (P2) • One of the things I appreciate about this (opera) is there are different perspectives in the family; so, the Veteran has his or her perspective, it also affects the partner or spouse, can hurt when you don’t understand what’s going on; kids can also feel that, it affects the entire family. (P1) • I could really relate to the spouse to the fear in walking around, setting him off, trying to help and be supportive. (P2) • The homeless aspect – it’s never giving up; no one wants to be homeless. They belong to the community. (P1)
Subtheme: Sharing available resources for Veterans and Families	• I really appreciate showing that there are so many services available for Veterans, either offered through the VA or if people aren’t eligible, there are so many people who want to give back to that specific community. The only thing that’s hard is getting Veterans to want those services […] Many times we see the Veterans waiting until they hit rock bottom, waiting until that substance abuse turns into homelessness, turns into suicidality, and then turns into that isolation when they are alone, and push everyone they care about away. So oftentimes we have to meet them where they are at, give them a sense of hope and remind them that there are services available. (P1) • Many times we see Veterans waiting until they hit rock bottom. Substance abuse turns into homelessness, turns into suicidality, isolation, pushes everyone they care about away. Meet them where they are at—give them a sense of hope—services are available—focus on meeting them where they are at. (P1) • I can’t expect someone to go if I’m not willing to put in the same kind of work myself. (P1) • Talking to a wife that worked at the VA, her husband was put in the VA mental ward. He died by suicide. I’m so grateful that here there is a program that helps. (P2) • It affects the entire family. But somewhere along the line, with the Veterans I’ve worked with there’s a catalyst there. They meeting somebody, it can be another Veteran—oftentimes from an older generation—that they connect with. And once you can get into that space where you start to learn about what PTSD is, it starts to demystify a lot of these things that people experience. It’s like that cliché that “knowledge is power.” One of the first steps is knowing and understanding that step. And one of the ways civilians can be helpful, understanding a little bit about PTSD, what the reactions can be and learning about the Veteran experience. And you do that by talking to Vets and listening to their experience. (PI)

## Results

Across performances, there were 68 audience members, 34 performers (5 leads), and 5 production staff (*N* = 107). Discussion comments were made after the first performance by Veterans (male, *n* = 5), family members (male, *n* = 3, including one from the cast; female, *n* = 1); providers (male, *n* = 2, including 1 family member of a Veteran and 1 Veteran; female, *n* = 1), and cast members (*n* = 3). The second performance had discussion comments from Veterans (male, *n* = 2; female, *n* = 2), family members (male = 1; female, *n* = 2), a provider (male, *n* = 1, who was also a family member of a Veteran), cast members (*n* = 5), and production staff (*n* = 1). The composer joined the discussions following both performances.

Three main themes were identified: (1) *Struggling Post-Military Service*; (2) *Finding Hope*; and (3) *Healing through the Opera*. Notably, the live performances and post-discussions enhanced participants’ understanding of mental health through the diverse perspectives of Veterans, family members, providers, and cast members. In the following sections, audience quotes are noted as Male (M) or Female (F), and by Performance (P1, P2). Key quotes by theme/subtheme are included in Table 2, with select exemplar quotes included below.

### Struggling Post-Military Service

Struggling post-military service was a theme shared across Veterans in the audience and encompassed two dimensions: *Feeling misunderstood* and *Self-isolating behavior*. They expressed that depictions in the opera of hardship after service resonated with their personal experiences. One noted: “It [the opera] brings to life everything we struggle with. No matter how long you’ve been home, we constantly struggle with what being in combat, being in war, did” (M, P1). Additional parallels between the opera and Veterans’ experiences stimulated discussions about struggles with homelessness, PTSD, and substance abuse. One shared, “Separating from the military, it took me to some pretty dark places, often in homelessness and in substance abuse” (Host, P2). Like the opera, Veterans shared that struggles related to military service were ongoing (Table 2).

#### Feeling misunderstood

Feeling misunderstood was part of the struggles Veterans expressed experiencing after military discharge. Some participants worried about civilians’ perceptions of Veterans who returned from war. One Veteran shared: “It’s tough about getting out [of the military]; I resented it because I thought it turned me into this beast that a lot of people saw " (M, P1). Another Veteran reflected, “It’s a bitch really. People don’t understand what you have to keep doing in order to [have] ‘some enchanted evening’ [as depicted in the opera]” (M, P1). Feeling misunderstood was discussed as inherent to struggles of departing from military service.

#### Self-isolating behavior

Self-isolation from family, friends, and other Veterans was another feature of the struggles Veterans experienced post-military life. Veterans in the audience identified with the self-isolating behavior and sense of aloneness by the opera’s main character. “I think that the thing of him [opera character] saying ‘I want to be alone, I need to be alone, but I hate to be alone’ that really resonated with me” (M, P1). In addition to recognizing their own withdrawal from family members, Veteran audience members identified isolating behavior of their peers and how that impacts their mental health care utilization: “Unfortunately, our friends and former Veterans don’t take advantage of [resources]. They want to isolate, to be alone. It’s not the way to heal” (M, P1). Another Veteran offered that a contributing factor might be “survivor’s guilt—just wanted to touch on that a lot of Veterans isolate themselves, too, from other Vets because of that” (M, P1). Feeling misunderstood and self-isolating while simultaneously feeling alone were noted as key aspects of Veterans’ struggles post-military service.

### Finding Hope

Despite the struggles that Veterans and other audience members described, many comments affirmed that “There can be a path forward” (Cast, P2) as it relates to finding hope and looking forward to a new future post-military service. Important components include understanding/being understood for both family members and Veterans and ensuring that Veterans experience a sense of community post-military service. One family member commented: “It’s very hard for other people to understand why you hang in there. I loved how that was presented [in the opera]. It’s very important for people to understand” (F, P1). A Veteran noted that it was “a real relief to know that you [the composer] do understand and are sharing it with the community” (F, P2). A member of the production team stated: “What I appreciated about this is that it really is about community, and being aware of the people around you, and knowing who you can go to, and understanding that there are resources out there” (P2). The theme of “finding hope” extended beyond the military service-related experiences presented in the opera.

### Healing Through the Opera

The “Veteran Journeys” opera impacted many production team and audience members by reminding them of their service- and nonservice-related experiences with trauma and healing. A Veteran cast member shared: “I think that honoring the service and honoring the experience of what happens when you return—through music— can aid in healing” (P1). A Veteran audience member expressed gratitude for the use of opera to amplify Veteran’s experiences: “I love the way this is going–thankful for putting music to the vibe, the whole story” (M, P1). While some reflected on the usefulness of music and art in healing, others noted that healing was also fostered in community with one another. For instance, a production team member reflected on their trauma from the loss of a family member and described how being part of the production and post-discussion process stimulated their healing: “Coming here though was finding a support system and learning how to ask for it” (P2).

Some cast members also saw themselves as part of the audience members’ healing process, noting the healing value of expressing others’ lived experiences through art. One cast member described their involvement in the opera as: “Marrying two of my passions, making an impact and also singing” (P2). Another cast member stated: “[Audience members] You hear a pretty song, you [cast members] think it spoke to the problem.” (P1). The added benefit of artists as healers was additionally described by a cast member as follows: “If you can heal through art, beauty and song and story—I just love that we may be able to contribute to that through telling this story” (P2). One noted the influence of live versus virtual performance: “I recorded this during the [COVID-19] lockdown, and performing it now live, is a completely different experience” (P2).

The “Veteran Journeys” opera promoted healing for Veterans and cast members through relatability to storyline, music, and development of community. The healing benefits were further categorized into three subthemes: (1) *Addressing difficult societal issues among Veterans*; (2) *Enhancing empathy towards Veterans and military families*; (3) *Sharing available resources for Veterans and families*.

#### Addressing difficult societal issues among Veterans

The opera, as an artistic platform, brought healing by addressing difficult societal issues among Veterans. For example, among several participants who described the opera as a healing mechanism (Table 2), one cast member reflected on how the opera brought healing to their experiences of PTSD: “To be able to sing an opera that is about something (PTSD) similar to what I’m going through, for people to be able to see it […] this is a way to bring forth more healing” (P2). Another cast member reflected on hardships experienced by Veterans and promoting healing through the opera: “I’ve spent some time with Veterans, and it’s a huge responsibility for us, as a society, to honor that and also to help [them] heal” (P1).

#### Enhancing empathy towards Veterans and military families

The opera was seen as enhancing empathy towards Veterans and military families and promoting healing. While many recognized the hardships of military service, by giving insight into post-discharge struggles the opera enhanced empathy. A participant who had family in the military shared: “This opera helped me open my eyes to see what Veterans had gone through and what they experienced” (F, P2). The inclusion of Veterans’ families’ experiences was noted to expand empathy towards military families. A provider reflected: “One of the things I appreciate about this opera is there are different perspectives in the family” (M, P1). A provider family member related to the story of the wife of the Veteran with PTSD: “I could really relate to the spouse” (F, P2). Another noted: “The homeless aspect – it’s never giving up” (F, P1). Thus, a key subtheme was promoting empathy by portraying effects of military service on individuals and families.

#### Sharing available resources for Veterans and families

Sharing resources through the opera was an action-oriented way of promoting healing. In addition to a representative from a well-being center for Veterans stating in the post-discussion, “We will help you get in touch with any kind of resource that is a good fit” (P1), a provider/Veteran reflected on the usefulness of the opera in highlighting resources for Veterans: “I really appreciate showing that there are so many services available for Veterans” (F, P1). Another provider reflected on how providers can support recovery: “Meet them where they are at. Give them a sense of hope. Services are available” (M, PI). The services brought forth in the opera and the post-discussion included those for family members of Veterans—especially for mental health care because, as one provider emphasized, “It affects the entire family” (M, PI). For example, a provider shared: “Talking to a wife that worked at the VA, her husband died by suicide. I’m so grateful that, here, there is a program that helps.”

## Discussion

This qualitative study described the perspectives of multiple stakeholders (Veterans, families, providers, general audience members, cast and crew) after live opera performances based on true stories of Veterans with histories of PTSD and homelessness, with post-discussions facilitated by a Veteran. Major themes centered on participants sharing personal experiences, including homelessness and PTSD, reflecting on VA resources, and engaging with experiences of Veterans through art.

There were noteworthy distinctions between the comments shared in the post-discussions of live-streamed, pre-recorded performances that we previously reported (Bilder et al., 2022) and the post-discussions for the live performances reported in the present study. For the live-streamed, pre-recorded productions, the post-discussions were not interactive. Concerns were expressed about witnessing trauma/abuse; the opera being more supportive of the VA relative to Veterans; technical issues, such as vocalizations being hard to understand; and interest in post-discussions facilitated by Veterans. Positive comments from the live-streamed audiences centered on the music, performance, cast, and learning about Veteran experiences, related stigmas, and hope (Bilder et al., 2022). In contrast, in the live performances reported here, the post-discussion exchanges among audience members, cast, and the Veteran facilitator were interactive. Post-discussion comments were also more Veteran- and family-centered. Challenges and struggles during post-military experiences were shared by Veterans, family members, providers and cast members. Efforts to find hope through connection and services were discussed, along with ways the opera as an art form, can help support healing. This approach aligns with *Contact! Unload*, a Canadian research-based play that was performed by Veterans. In post-performance surveys, 96% of audience members agreed that using Veterans as actors was an important element of the play, and that the play helped audience members to better understand the need for support programs for Veterans (Belliveau & Nichols, 2017). These findings highlight the critical role of Veteran involvement in both performance and post-discussion facilitation, which appears to foster authentic dialogue, increase understanding, and create meaningful connections between Veterans, families, providers, and the broader community.

The diverse perspectives shared through this opera created meaningful exchanges among Veterans, family members, providers, audience members, and cast. Live performances proved particularly effective at encouraging personal storytelling and experience-sharing, creating an atmosphere similar to peer-facilitated “group therapy” (Mercier et al., 2023). This was evidenced by audience members exchanging meaningful quotes from the libretto, such as “We are not alone,” which highlighted their shared experiences. Our findings align with those from *The Shell Shock Performance* (Winn & Odell-Miller, 2018), which demonstrated how live theatre can increase awareness and shift public attitudes toward Veterans’ mental health concerns. Our findings are also consistent with those from a study of people experiencing homelessness (PEH) who participated in a therapeutic songwriting program and shared their songs with community members (Landless et al., 2025). That study reported similar interactions between participants and community members that included mutual sharing, support, and increased understanding of the PEH’s experiences.

Research has consistently demonstrated the positive effects of peer support and creative arts therapies on mental health recovery, including within VA settings (Chinman et al., 2012; Jewel et al., 2022; Mercier et al., 2023; Thomas et al., 2023), yet opera performances and peer-facilitated discussions remain understudied. In contrast to our prior study of the live-streamed, pre-recorded performances (Bilder et al., 2022), findings from the current study suggest that the in-person performance format combined with immediate peer-facilitated discussion effectively encouraged dialogue among Veterans, families, providers and cast. These findings suggest that integrating artistic engagement, collective reflection and dialogue, and sharing resources may help address ongoing challenges such as homelessness and PTSD/depression in military/Veteran populations (Moore et al., 2023; Tsai et al., 2021). These findings further align with previous research showing opera’s potential to increase mental health engagement (Agius & Agius, 2022; Skrine Jeffers et al., 2022) and contribute to the broader literature on creative arts therapies across clinical and community settings (Chilton et al., 2021; Vaudreuil et al., 2019; Wang et al., 2025).

Following a socioecological framework (Mango et al., 2018; Salihu et al., 2015) future research should examine three key areas: performance format (live versus pre-recorded and streamed), content (mental health and social factors), and context (audience composition, cast involvement, and peer facilitation). Additionally, studies should assess longer-term impacts of these performances, the specific effects of peer-facilitated discussions, and the outcomes of partnerships with Veteran-serving institutions in addressing mental health themes.

### Limitations

The post-discussions had a similar number of comments as online streaming (Bilder et al., 2022). In the present study, the composer and cast were present during the post-discussions, which could have prompted positive comments from audience members. However, individuals shared more personal traumatic experiences in-person following the live performances than during the post-discussions following the live-streamed recorded performances (Bilder et al., 2022). Verifying that analyses would be anonymous and peer facilitation by a Veteran could have facilitated more personal sharing. Further, this analysis was limited to two post-discussions in an academic auditorium with resources shared by providers and Veterans brought from a local VA. In addition, the 30-minute time period for post-discussion likely limited the possibilities for depth, breadth, and variety of content given the number of questions that were covered during the discussion. Finally, audience sizes were modest given COVID-19 policies that limited attendance, and the limited identity information gathered from participants may have negated nuanced understandings that could be connected to the impacts of larger societal systems on audience members’ lived experiences.

## Conclusion

Discussions facilitated by a Veteran peer suggested that live opera events may stimulate reflections by Veteran, family and cast members on trauma and homelessness, benefits and challenges in receiving services and sharing of audience and cast experiences of stress, trauma and recovery through art. While limited to two performances and descriptive analysis, the findings may have implications for research on the impact of live art events on experiences such as homelessness and PTSD with peer facilitation.
